# Brain stem herniation secondary to cerebrospinal fluid drainage in ruptured aneurysm surgery: a case report

**DOI:** 10.1186/s40064-016-1875-4

**Published:** 2016-03-01

**Authors:** You-Sub Kim, Sung-Hyun Kim, Seung-Hoon Jung, Tae-Sun Kim, Sung-Pil Joo

**Affiliations:** Department of Neurosurgery, Chonnam National University Hospital Biomedical Research Institute, Chonnam National University Hospital and Medical School, 42, Jebong-ro, Donggu, Gwangju, 501-757 Republic of Korea

**Keywords:** Subarachnoid hemorrhage, Lumbar drainage, Brain sag, Trendelenburg position

## Abstract

**Background:**

A lumbar drainage catheter is frequently placed intra-operatively to decrease fluid pressure on the brain in aneurysmal subarachnoid cases. In rare cases, this catheter placement can lead to intracranial hypotension, resulting in brain stem herniation termed “brain sag” and it can lead to neurological injury and may prove to be fatal. We present our patient with brain sag secondary to intraoperative lumbar drainage.

**Case description:**

A 56-year-old woman was admitted with a sudden onset of severe headache. A computed tomography (CT) scan revealed diffuse subarachnoid hemorrhage with ruptured anterior communicating artery aneurysm. After general anesthesia, a lumbar drainage catheter was placed intra-operatively to reduce pressure on the brain and 50 cc of CSF was removed during a 5-h period. Three to five days after operation, her neurologic symptoms became worse with an altered mental state and pupillary asymmetry. CT and magnetic resonance imaging (MRI) showed slit lateral ventricles, effacement of the cisterns and an elongated brain stem. After placing the patient in the Trendelenburg position, the patient rapidly recovered to her baseline neurologic state.

**Discussion:**

Typical complications of subarachnoid hemorrhage such as vasospasm or hydrocephalus also manifest as neurological deterioration, but their treatments differ greatly from those for brain sag. Thusly, it is important to distinguish between causes. Treatments such as lumbar or extra-ventricular drainage, induced hypertension or administration of mannitol must be stopped once brain sag is suspected. Also, care should be taken for typical imaging features of brain sag on CT or MRI scan. For brain sag, placing the patient in the Trendelenburg position can improve neurological status in a rapid fashion.

**Conclusions:**

Brain sag is a rare but serious condition and can be fatal if not rapidly diagnosed and treated. We therefore recommend including brain sag in the differential diagnosis, along with vasospasm, hydrocephalus or cerebral edema as part of possible complications following subarachnoid hemorrhage treatment. We hope our clinical and imaging data from this case study contribute to the correct diagnosis of brain sag, as its early detection is important.

## Background

During surgery to treat aneurysmal subarachnoid hemorrhage (SAH), neurosurgeons often place a lumbar drainage catheter or perform a ventriculostomy after general anesthesia to lower intracranial pressure (ICP). These procedures serve to allow the brain to relax as well as secure the surgical field and avoid retraction injuries.

Nevertheless, the loss of too much cerebrospinal fluid (CSF) precipitating mild to severe complications is a possibility associated with these maneuvers. Mild and self-limiting complications include the onset of postural headaches, persistent CSF leaks requiring epidural blood patches, nausea with vomiting, decreased vision, abducens nerve palsy or tinnitus, whereas severe complications could prove fatal as they are the result of brain stem herniation. Brainstem herniation as a complication of this specific maneuver is known as “brain sag”. Brain sag symptoms include altered mental status, pupillary asymmetry, and decerebrate posturing (Bloch and Regli 2003; Kelley and Johnson 2004; Roland et al. 1992). From brain computed tomography (CT) or magnetic resonance imaging (MRI), intracranial hypotension can manifest with diffuse meningeal enhancement, “brain sag” morphology, effacement of CSF cisterns or Arnold-Chiari malformation (Atkinson et al. 1998; Hochman and Naidich 1999; Savoiardo et al. 2010).

As mentioned above, most patients with intracranial hypotension experience self-limiting and mild symptoms as they can occur after transient lumbar puncture, continuous placement of a lumbar drainage catheter, traumatic brain injury, extraventricular drainage, or brain surgery from manipulating the dura. These may be related to the downward migration of brain resulting in traction of the dura mater, stimulating chemotactic areas and putting pressure on cranial nerves (Grant et al. 1991; Hochman and Naidich 1999; Miyazawa et al. 2003; Niedermuller et al. 2002; Wang and Schmidt 1997). For mild symptoms, bed rest without head elevation and adequate hydration with analgesics is recommended as initial management. Sometimes, however, an invasive treatment such as an epidural blood patch is required (Bezov et al. 2010).

On the other hand, intracranial hypotension with brain stem herniation and brain sag causes an altered mental status and signs of herniation after intraoperative lumbar drainage, especially in aneurysmal SAH patients (Connolly et al. 1997; Samadani et al. 2003). This may lead to permanent neurologic deficit or it may be fatal (Alaraj et al. 2011; Komotar et al. 2005; Samadani et al. 2003).

In this report, we present our patient with aneurysmal SAH who suffered brain stem herniation secondary to intraoperative lumbar drainage and review the clinical symptoms with radiologic findings.

## Case description

**Fig. 1:**
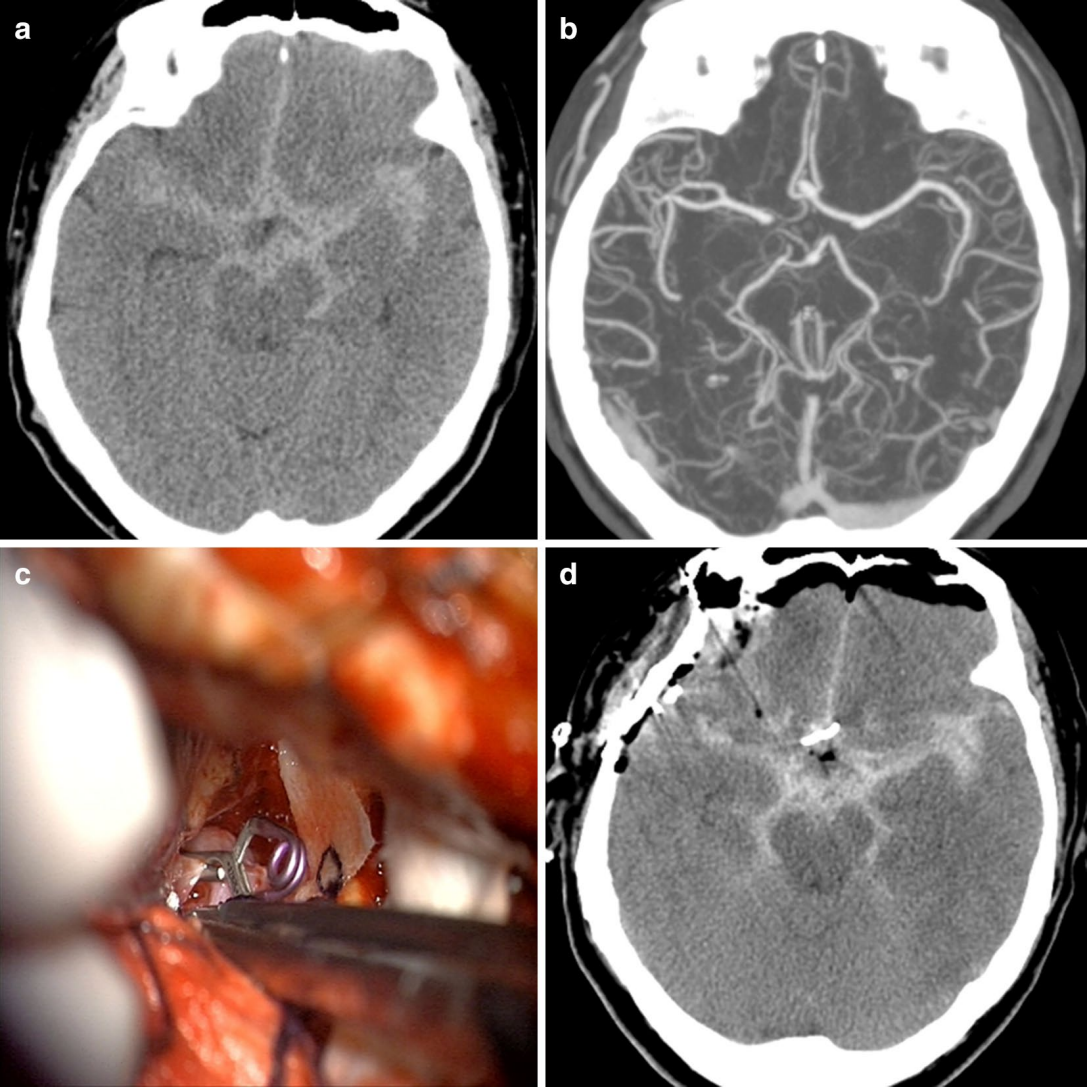
Axial non-contrast CT scan showed diffuse SAH in the basal cisterns, anterior interhemispheric fissure and sylvian fissures (**a**). CT angiography demonstrated ruptured aneurysm of the anterior communicating artery (**b**). Intraoperative photograph and post-operative CT scan showed the clipped aneurysm without any complications (**c**, **d**)

A 56-year-old woman with no significant previous medical conditions was admitted after a sudden onset of severe headache (Hunt-Hess classification 2 and WFNS grade 2). A brain CT scan showed diffuse SAH without intraventricular hemorrhage or hydrocephalus (Fisher grade 3). A subsequent CT angiography demonstrated a 5-mm-sized anterior communicating artery aneurysm. We decided to perform craniotomy to repair the aneurysm. A right fronto-temporal craniotomy was done and the ruptured aneurysm was clipped via the pterional approach without any observed complications (Fig. 1). After general anesthesia, lumbar drainage catheter was placed to relax the brain and 50 cc of CSF was removed during a 5-h period. The drainage catheter was clamped at the end of the operation. The amount of CSF removed during the perioperative period is described in Table 1. Post-operatively, the patient was able to open her eyes in response to speech and obey commands without significant weakness in the extremities.

**Table 1:** Amount of CSF drainage during perioperative period

	Total CSF drainage^a^	Lumbar drain	Extraventricular drainage
During operation	50 cc	50 cc	–
Postoperative Day 1	0	Clamp	–
Day 2	0	Clamp	–
Day 3	0	Remove	–
Day 4	150 cc	–	150 cc
Day 5	16 cc	–	16 cc → Clamp
Day 6	0 cc	–	Clamp
Day 7	60 cc	–	Open → 60 cc

^a^Total CSF drainage means the sum of lumbar drainage and extraventricular drainage

**Fig. 2:**
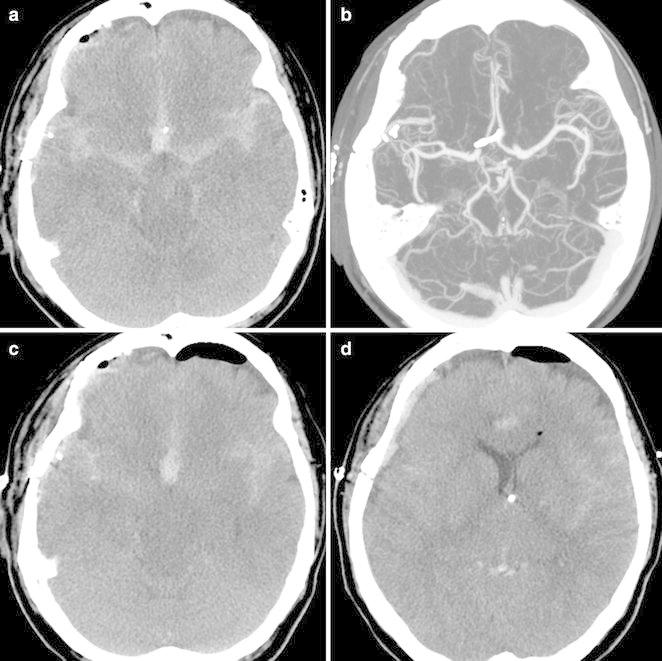
On post-operative Day 3, (**a**, **b**) head CT scan and CT angiography revealed no evidence of re-bleeding, hydrocephalus or vasospasm but showed an elongated midbrain. On post-operative Day 5 (**c**, **d**), head CT scan showed small lateral ventricles and effacement of the cisterns

Day 3 after operation, the patient developed drowsiness. CT angiography was performed but there was no evidence of re-bleeding, vasospasm, hydrocephalus or infarction (Fig. 2a, b). A clinical diagnosis of vasospasm was made and hyperdynamic therapy was performed accordingly with norepinephrine, albumin and plasma expander maintaining systolic blood pressure over 170 mmHg.

On post-operative Day 4, she showed progressive neurological deterioration manifested by her stuporous mental status. We decided to monitor the ICP and a ventriculostomy was performed. The pressure was low, ranging from 5 to 10 cm H_2_O and 150 cc of CSF was removed during the next 24 h.

The next day (operative Day 5), her neurologic conditions worsened and she was noted to have decerebrate posture was along with pupillary asymmetry. The patient was intubated and a CT scan was performed. This scan showed small lateral ventricles, effacement of the cisterns and elongated brain stem suggesting brain sag (Fig. 2c, d). Hyperdynamic therapy and mannitol administration were immediately discontinued and the extraventricular drainage was clamped, followed by the patient being placed in the Trendelenburg position. She showed rapid recovery to her baseline neurologic conditions within 9 h.

**Fig. 3:**
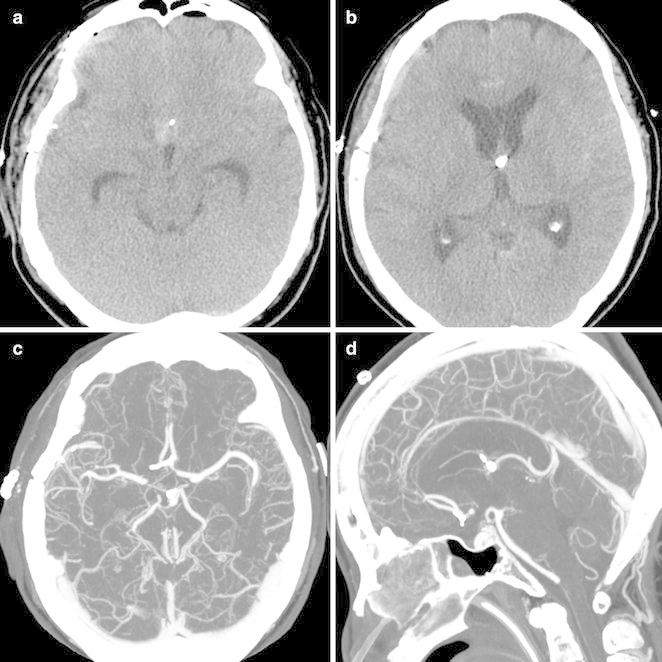
On post-operative Day 7, the hematoma was nearly absorbed and ambient cistern was visible (**a**). But ventricles were enlarged compared with the previous CT scan (**b**) and CT angiography demonstrated significant vasospasm at both distal portions of the anterior cerebral arteries (**c**, **d**)

**Fig. 4:**
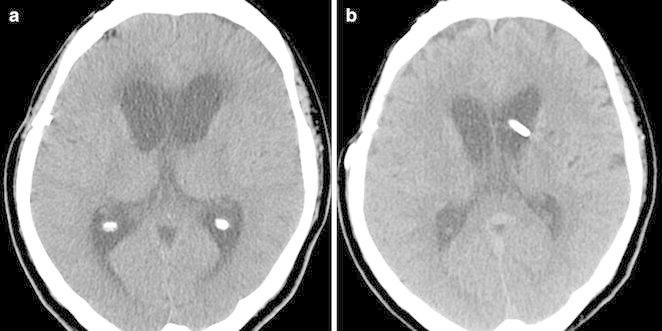
A follow-up CT scan showed a communicating hydrocephalus (**a**) and a ventriculo-peritoneal shunt were placed 3 months after surgery (**b**)

After this initial improvement, her neurologic condition deteriorated again as exhibited by a stuporous mental state 7 days after surgery. CT angiography was performed and demonstrated significant diffuse vasospasm. Also, the ventricles were enlarged compared with the previous CT scan (Fig. 3). It was decided to resume hyperdynamic therapy and drainage of the CSF. The patient recovered soon and was discharged 1 month after surgery. A ventriculo-peritoneal shunt was performed to treat hydrocephalus secondary to SAH 3 months after the operation (Fig. 4).

## Discussion

In ruptured aneurysm surgery, brain relaxation is necessary to expose the Circle of Willis. There are a number of procedures available to achieve this such as preoperative or intraoperative ventriculostomy and placement of a lumbar drainage catheter (Bailes et al. 1990; Paine et al. 1988; Samson 1993). Between these, lumbar drainage is preferred because it is associated with fewer complications than ventriculostomy. The common complications of ventriculostmy are bleeding, infection and CSF leakage (Hasan et al. 1989, 1991). In our hospital, intraoperative placement of a lumbar drainage catheter (with a 16-gauge needle) has been routinely performed in SAH patients except when space-occupying lesions are detected from preoperative brain imaging scan, there is intraventricular hemorrhage or intracerebral hematoma is detected.

Although intraoperative CSF drainage via lumbar drainage catheter is the preferred technique, in rare cases it can cause intracranial hypotension leading to brain stem herniation and brain sag after treating the aneurysmal SAH (Alaraj et al. 2011; Komotar et al. 2005; Samadani et al. 2003). According to the literature, there have been cases of spontaneous intracranial hypotension and its clinical manifestations are well recognized (Ferrante et al. 2004; Pakiam et al. 1999; Schievink 2008; Schievink et al. 1996). But brain sag after intraoperative lumbar spinal drainage during ruptured aneurysm surgery is a rare complication and only a handful of cases have been reported. Because of this unfamiliarity, brain sag may easily be misdiagnosed.

Komotar et al. (2005) reported 11 patients with brain sag after craniotomy. Most patients experienced this syndrome on post-operative Day 3 and never after post-operative Day 5. In our case, we considered the patient’s clinical presentation a result of increased intracranial pressure (IICP) around the brain stem and due to clinical vasospasm. However, the CT scan performed on the third day after the operation showed an elongated midbrain and effacement of the cisterns, symptoms clearly pointing to brain sag. As the patient underwent ventriculostomy for ICP monitoring and hyperdynamic therapy, her neurologic condition deteriorated. On post-operative Day 5, all of treatments, such as the administrations of mannitol, and inotropics, as well as CSF drain, were stopped. The patient improved soon after placement in the Trendelenburg position.

Common and well-known complications after SAH include hydrocephalus, vasospasm, infarction and cerebral edema. These often require invasive procedures including angiography or ventriculostomy which lead to additional risks of post-operative morbidity. Care should be taken not to misdiagnose brain sag if these patients present with an altered mental status, pupillary changes, or a decerebrate posture after lumbar CSF drainage (for example performed during aneurysmal surgery). Intracranial hypotension has also been reported in various surgical intracranial procedures without lumbar drainage. However, we conclude that lumbar drainage of the CSF was responsible for the hypotension in this case because the patient became progressively aggravated after intraoperative lumbar drainage without any surgical intervention.

**Fig. 5:**
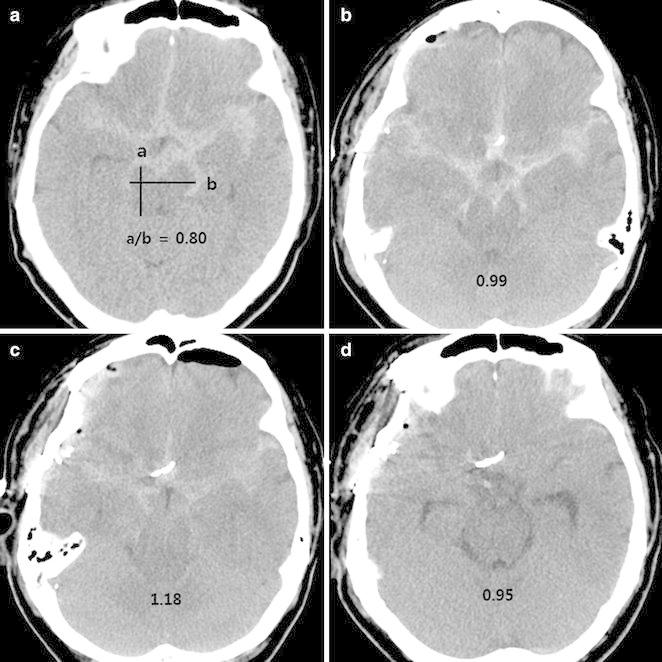
Schema of the sag ratio calculated with **a** maximal AP diameter and **b** maximal bipeduncular diameter at the level of midbrain (**a**). Axial CT scan performed before surgery, Day 3, 5 and 7 after operation (**b**–**d**). The sag ratio of each was 0.80, 0.99, 1.18 and 0.95, respectively. CT scan on post-operative Day 5 (**c**) showed effacement of the sylvian and prepontine cisterns as well as an elongated midbrain

Komotar et al. presented three diagnostic criteria for brain sag: a CT scan showing (1) effacement of the cisterns and an elongated midbrain, (2) rapid improvement from placement in the Trendelenburg position, and finally (3) mid-sagittal MR or CT images showing descent of the brain stem. Komotar et al. introduced the “sag ratio”, which is calculated from the measurement of the maximal AP distance and the maximal bipeduncular distance of the midbrain. Komotar et al. found the normal “sag ratio” to be about 0.9, and in the presence of brain sag the ratio was 1.18 (from the study of 11 patients) (Komotar et al. 2005). In our case, the “sag ratio” before surgery, Day 3, 5 and 7 post-surgery was 0.80, 0.99, 1.18 and 0.95 respectively (Fig. 5).

**Fig. 6:**
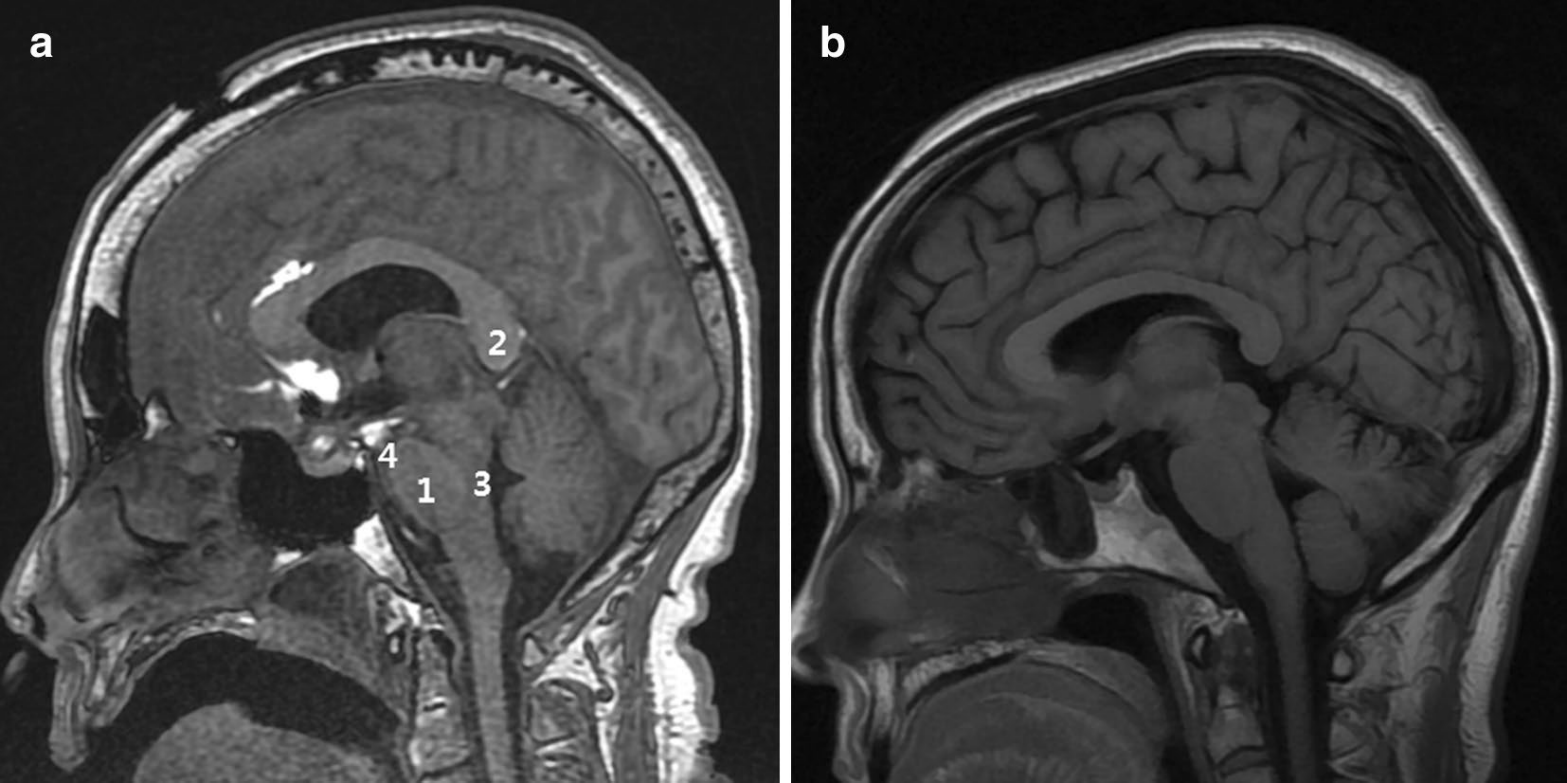
T1-weighted mid-sagittal MRI performed on post-operative Day 5 (**a**). Compared with age-matched normal brain MRI (**b**), it demonstrated *1* an oval shaped pons, *2* downward migration of the splenium, *3* vertical shaped anterior wall of the 4th ventricle and *4* narrowing of the interpeduncular and prepontine cisterns

The patient’s brain MRI was checked to confirm brain sag on post-operative Day 5. There were significant differences compared with age-matched normal MRI, especially on the mid-sagittal plane: (1) an elongated or oval shaped pons, (2) narrowing of the interpeduncular and prepontine cisterns, (3) elongation and downward migration of the splenium, and (4) a more vertical direction of the anterior wall of the 4th ventricle (Fig. 6).

Four studies have previously provided data on the clinical features and treatments of brain sag in SAH patients (Alaraj et al. 2011; Kawahara et al. 2013; Komotar et al. 2005; Samadani et al. 2003). The majority of patients showed improvement of symptoms after placement in the Trendelenburg position, just as we had observed in the present case. In these studies, invasive procedures such as placing an epidural blood patch and exploratory craniotomy were needed to stem persistent CSF leaks or when a space-occupying lesion was detected (see Table 2). To date, aside from a few case reports, there has not been a prospective study about brain sag after lumbar CSF drainage performed during aneurysmal surgery. Given the unlikelihood of such a prospective study being conducted, it is our opinion that a future study focusing on the amount of CSF drainage that brings about brain sag should be considered.

**Table 2:** Summary of the literature search on diagnosis and treatment for brain sag in SAH

References	No. of patients	Clinical manifestations	Brain image (CT or MRI)	Amount of CSF removed intraoperatively (cc)	Treatment	Permanent neurologic deficit
Samadani et al. (2003)	2	Drowsiness Cranial N. deficits	Epidural hematoma Effaced cisterns Brain stem infarction	50 cc, 63 cc	Epidural blood patch, Surgery^a^	1^b^
Komotar et al. (2005)	11	Decrement of GCS Pupillary asymmetry	Oblonged brain stem Effaced cisterns	Not documented	Trendelen-burg	2^c^
Alaraj et al. (2011)	5	Decerebrate posture Pupillary change	Oblonged brainstem	30 cc	Trendelen-burg	1(dead)
Kawahara et al. (2013)	11	Anisocoria Mental status decline	Oblonged brainstem Effacement of basal cisterns	Not documented	Trendelen-burg	Not documented
Kim et al. (2016) (Present study)	1	Decreased mentality Pupillary asymmetry	Downward migration of splenium Oval shaped pons	50 cc	Trendelen-burg	0

^a^Surgical evacuation was needed in one patient due to epidural hematoma with mass effect and midline shift. Another patient required epidural blood patch for persistent CSF leak

^b^The patient with brain stem infarction failed to respond to epidural blood patch

^c^Patients with Hunt and Hess grade IV

## Conclusions

Brain sag after intraoperative lumbar drainage in SAH patients is a severe form of intracranial hypotension characterized by neurologic deteriorations and signs of herniation such as pupillary change and/or decerebrate posturing. It is a rare condition but it may lead to severe permanent neurologic deficit or death, and as such, brain sag should be included in the differential diagnosis, along with vasospasm, hydrocephalus, or cerebral edema, in the post-operative aneurysmal SAH patient with neurological and/or mental status changes. In conclusion, we hope our clinical and imaging features contribute to the database of knowledge in diagnosis of brain sag, as its early detection is important.
